# Seroprevalence of Kaposi Sarcoma–associated Herpesvirus and Other Serologic Markers in the Brazilian Amazon

**DOI:** 10.3201/eid1504.081488

**Published:** 2009-04

**Authors:** Maria C. Nascimento, Laura M. Sumita, Vanda U. Souza, Helen A. Weiss, Juliane Oliveira, Melissa Mascheretti, Mariana Quiroga, Rodrigo A.R. Vela, Ester Sabino, Claudio S. Pannuti, Philippe Mayaud

**Affiliations:** Universidade de São Paulo, São Paulo, Brazil (M.C. Nascimento, L.M. Sumita, V.U. Souza, J. Oliveira, M. Mascheretti, M. Quiroga, R.A.R. Vela, E. Sabino, C.S. Pannuti); London School of Hygiene and Tropical Medicine, London, UK (M.C. Nascimento, H.A. Weiss, P. Mayaud)

**Keywords:** Virus, Kaposi sarcoma, herpesvirus, human herpesvirus type 8, Amerindian, epidemiology, blood donors, Amazon region, Brazil, dispatch

## Abstract

To determine the presence of Kaposi sarcoma–associated herpesvirus (KSHV) and other serologic markers, we tested serum specimens of 339 Amerindians, 181 rural non-Amerindians, and 1,133 urban blood donors (13 Amerindians) in the Brazilian Amazon. High KSHV seroprevalence in children and inverse association with herpes simplex virus type 2 indicates predominant nonsexual transmission among Amerindians.

Kaposi sarcoma–associated herpesvirus (KSHV) is the cause of Kaposi sarcoma (KS) and certain lymphoproliferative diseases (*1*). KSHV seroprevalence is low (<5%) in most Western populations (*1*) and reaches 50% in some African populations (*2*), mirroring KS incidence rates (*3*). However, the highest KSHV seroprevalences worldwide (>80% in adults) have been reported in Amerindian tribes from the Amazon regions of Brazil (*4*,*5*) and Ecuador (*6*), despite the apparently low KS incidence in these populations (*7*). KSHV is thought to be transmitted through saliva between young siblings in disease-endemic areas such as French Guiana (*8*) or Africa (*9*), whereas sexual transmission in low-prevalence countries occurs within risk groups such as men who have sex with men (*10*). Modes of transmission have not been clearly determined in Amerindian populations.

## The Study

We conducted a cross-sectional study to investigate the seroprevalence and factors associated with KSHV infection in Amerindian and non-Amerindian populations living in 2 regions of the Brazilian Amazon: a remote rural region of Para State (Mapuera, on the banks of the Trombetas River) and Manaus, the capital city of Amazonas State (Figure). Serologic markers of fecal–oral (hepatitis A virus [HAV]), blood-borne (hepatitis B and C viruses [HBV, HCV]) and sexually transmitted infections (*Treponema pallidum* [syphilis] and herpes simplex virus type 2 [HSV-2]) were used as proxies to identify possible routes of KSHV transmission in these populations.

**Figure:**
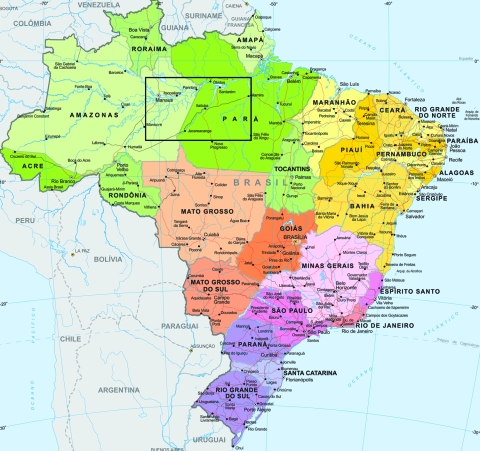
Map of Brazil showing study area (black box) in Amazonas (Manaus) and Para (Mapeura region) States. Printed with permission of the Instituto Brasileiro de Geografia e Estatística.

A convenience sample of unselected Amerindians and non-Amerindians living in the Mapuera area and a consecutive sample of nonpaid first-time blood donors from the Manaus blood bank (HemoAm) consented to collection of blood samples, as previously reported (*4*,*11*) Ethical approval was obtained from the institutional review board of HemoAm, the ethical board of the Brazilian Ministry of Health, and the ethics committee of the London School of Hygiene and Tropical Medicine.

In the absence of a definitive test to determine KSHV infection, all serum specimens were tested by using a previously validated in-house whole-virus KSHV ELISA (*12*) and 2 immunofluorescence assays (IFAs) that detected antibodies against lytic (IFA-lytic) and latent-associated nuclear antigens (IFA-LANA) (*12*). KSHV infection was defined as positivity by any of these serologic assays. Serum specimens were also tested for the agent of syphilis by using a *T. pallidum*–specific assay (Enzygnost Syphilis; Dade Behring, Marburg, Germany); for HSV-2 antibodies by using the type-specific HerpeSelect gG2 ELISA (Focus Technologies, Cypress Hill, CA, USA), with a higher cut-off (>3.5) to increase specificity (*13*); and for HAV antibodies by using BioELISA HAV (Biokit, Barcelona, Spain). Presence of HBV anti-core antibodies was determined by using Ortho HBc ELISA (Ortho Diagnostics, Raritan, NJ, USA) in Mapuera serum specimens and Hepanostika anti-HBc Uni-Form (Organon-Teknika, Boxtel, the Netherlands) in Manaus serum specimens. HCV antibodies were detected by using Ortho HCV 3.0 ELISA (Ortho Diagnostics) in Mapuera serum specimens and Murex Anti-HCV version 4.0 ELISA (Murex Biotech S.A., Kyalami, South Africa) in Manaus serum specimens.

KSHV seroprevalence was calculated separately for men and women and directly age-standardized to the Mapuera Amerindian population. The risk associated with KSHV infection was estimated with prevalence ratios (PRs) and 95% confidence intervals (CIs), adjusted for sex and age group (18–24 years, 25–34 years, and >35 years for the blood donor population; 0–9 years, 10–17 years, 18–24 years, 25–34 years, and >35 years for both Mapuera populations). The associations of KSHV with sociodemographic variables, indicators of socioeconomic status, and other serologic markers were estimated with odds ratios (ORs) and 95% CIs. Variables associated with a significant increased risk for KSHV (p<0.05) in univariable analysis were included in a multivariable logistic regression model adjusted for age and sex.

We recruited 339 Amerindians (median age 22 years, interquartile range [IQR] 13–37 years; 57.5% female) and 181 non-Amerindians (median age 17 years, IQR 9–35 years; 58.6% female) in the Mapuera communities and 1,133 blood donors (median age 25 years, IQR 21–32 years; 22.9% female) in Manaus. The blood donor population had a similar age distribution to that of the adult population in Manaus in the 2000 regional census (*14*).

Among Mapuera Amerindians, KSHV seroprevalence was 65.0% in those 0–9 years, increasing to 92.9% in those >35 years. In contrast, among Mapuera non-Amerindians, KSHV seroprevalence was 9.8% in those 0–9 years of age, increasing to 50.0% in those >35 years of age. Among blood donors, KSHV seroprevalence was 31.3% in those >35 years of age and 53.8% in the 13 who were of Amerindian descent. After age standardization, KSHV seroprevalence remained lower among Mapuera non-Amerindians (30% and 27% among men and women, respectively) and blood donors (16% and 23%, respectively) than among Mapuera Amerindians. When results were compared with those of the Mapuera Amerindians, the age-and sex-adjusted PRs were 0.35 (95% CI 0.28–0.45) and 0.59 (95% CI 0.56–0.63) in Mapuera non-Amerindians and blood donors, respectively.

In each population, KSHV seroprevalence was slightly higher among females, and increased with age (p for trend <0.001) in Mapuera Amerindians and non-Amerindians, but not among (adult) blood donors (Table 1). KSHV seroprevalence varied little with house crowding (socioeconomic indicator), and hepatitis infections, but was associated with HSV-2 infection in non-Amerindians (OR 4.2, 95% CI 2.1–8.5) and blood donors (OR 1.3, 95% CI 1.0–1.7). In Amerindians, KSHV infection was not associated with HSV-2 in univariable analysis (OR 0.7, 95% CI 0.3–1.9).

**Table 1:** Seroprevalence of KSHV among 3 populations in the Brazilian Amazon*†

Variables	Mapuera Amerindians, n = 339†			Mapuera non-Amerindians, n = 181†			Manaus blood donors, n = 1,133†	
	% KSHV positive (no. tested)	OR (95% CI)		% KSHV positive (no. tested)	OR (95% CI)		% KSHV positive (no. tested)	OR (95% CI)
Sex								
Male	79.2 (144)	1		26.7 (75)	1		28.6 (874)	1
Female	82.6 (195)	1.2 (0.7–2.1)		27.4 (106)	1.0 (0.5–2.0)		34.4 (259)	1.3 (1.0–1.7)
p value		0.4			0.1			0.08
Age group, y								
0–9	65.0 (43)	0.1 (0.05–0.4)		9.8 (51)	0.1 (0.03–0.3)		–	–
10–17	70.0 (93)	0.2 (0.07–0.4)		22.5 (40)	0.3 (0.1–0.7)		–	–
18–34	86.5 (104)	0.5 (0.2–1.3)		27.3 (44)	0.4 (0.1–0.9)		29.6 (916)	0.9 (0.7–1.3)
>35	92.9 (99)	1		50.0 (46)	1		31.3 (217)	1
p for trend		<0.001			<0.001			
Crowding‡								
1–2	93.7 (16)	1		55.6 (9)	1		32.6 (175)	1
3	91.3 (23)	0.7 (0.06–8.4)		33.3 (15)	0.4 (0.07–2.2)		29.9 (941)	0.9 (0.6–1.2)
>4	79.7 (300)	0.3 (0.03–2.0)		24.8 (145)	0.3 (0.07–1.3)		6.2 (16)	0.4 (0.2–1.0)
p value		0.1			0.1			0.1
Ethnicity								
African	–	–		–	–		29.6 (743)	1
Caucasian	–	–		–	–		30.5 (308)	1.0 (0.8–1.4)
Indigenous	100 (339)	–		–	–		53.8 (13	2.8 (0.9–8.3)
Other	–	–		100 (181)	–		25.8 (66)	0.8 (0. 5–1.5)
p value								0.08
Hepatitis A virus								
Negative	83.3 (6)	1		12.5 (16)	1		42.9 (7)§	1
Positive	81.1 (333)	0.9 (0.1–7.5)		28.5 (165)	2.8 (0.6–12.7)		28.6 (154)§	0.5 (0.1–2.5)
p value		0.9			0.2			0.4
Hepatitis B virus								
Negative	81.6 (315)	1		32.0 (75)§	1		30.2 (1,075)	1
Positive	73.9 (23)	0.6 (0.2–1.7)		53.3 (15)§	2.4 (0.8–7.5)		25.0 (56)	0.8 (0.4–1.4)
p value		0.4			0.1			0.4
Hepatitis C virus								
Negative	81.0 (338)			36.0 (90)†			29.9 (1,129)	1
Positive	0			0			25.0 (4)	0.8 (0.1–7.5)
p value								0.8
HSV-2								
Negative	81.5 (314)	1		18.1 (127)	1		27.8 (715)	1
Positive	76.0 (25)	0.7 (0.3–1.9)		48.1 (54)	4.2 (2.1–8.5)		33.2 (406)	1.3 (1.0–1.7)
p value		0.5			<0.001			0.06
*Trepomena pallidum*								
Negative	81.0 (338)	–		26.3 (171)	1		29.9 (1,122)	1
Positive	0			40.0 (10)	1.9 (0.5–6.9)		36.4 (11)	1.2 (0.6–2.3)
p value					0.3			0.7

*Seroreactivity by any serologic assay, whole virus. KSHV, Kaposi sarcoma–associated herpesvirus; OR, odds ratio; CI, confidence interval; HSV-2, herpes simplex virus type 2. †Some figures do not add up to the total because of missing data. ‡Number of residents living in the house. §Only a random subsample tested.

In multivariable analysis (Table 2), KSHV infection remained associated with female sex among blood donors (age- and sex-adjusted OR [aOR] 1.3, 95% CI 1.0–1.7), and increased significantly with age in both Mapuera populations (p for trend <0.001). KSHV infection was associated with HSV-2 infection among Mapuera non-Amerindians (aOR 2.7, 95% CI 1.2–6.5) and Manaus blood donors (aOR 1.3, 95% CI 1.0–1.6), but was inversely associated with HSV-2 infection in Mapuera Amerindians (aOR 0.3, 95% CI 0.1–0.9).

**Table 2:** Multivariable analysis of risk factors for KSHV infection among 3 populations in the Brazilian Amazon*

Variables	aOR (95% CI)		
	Mapuera Amerindians, n = 339	Mapuera non-Amerindians, n = 181	Manaus blood donors, n = 1,133
Sex			
Male	1	1	1
Female	1.2 (0.7–2.2)	1.0 (0.5–2.1)	1.3 (1.0–1.7)
p value	0.5	0.9	0.08
Age group, y			
0–9	0.1 (0.05–0.4)	0.1 (0.04–0.3)	
10–17	0.2 (0.07–0.4)	0.3 (0.1–0.7)	
18–34	0.5 (0.2–1.2)	0.4 (0.1–0.9)	0.9 (0.7–1.3)
>35	1	1	1
p value	<0.001	<0.001	0.6
HSV-2			
Negative	1	1	1
Positive	0.3 (0.1–0.9)	2.7 (1.2–6.5)	1.3 (1.0–1.6)
p value	0.03	0.02	0.09

*Seroreactivity by any serologic assay (whole virus ELISA, IFA-LANA, or IFA-lytic) in multivariable analysis. KSHV, Kaposi sarcoma–associated herpesvirus; IFA-LANA, immunofluorescence assay that detected latent-associated nuclear antigens; IFA-lytic, IFA that detected lytic-associated nuclear antigens; aOR, age- and sex-adjusted odds ratio; CI, confidence interval; HSV-2, herpes simplex virus type-2.

## Conclusions

Our data confirm the high KSHV seroprevalence observed among Amazonian Amerindian populations (*5*,*7*). However, the inclusion of convenience samples of remote populations and first-time blood donors, who may not necessarily be representative of the adult general population and notably exclude persons who report a range of potentially high-risk behavior for sexually transmitted and blood-borne infections, may have limited the generalizibility of our findings. High KSHV seroprevalence combined with an apparent lack of KS development among Amerindian populations support the theory of genetic predisposition to KSHV acquisition, as hypothesized for other Amazonian populations, in whom segregation genetic analysis has suggested that an unidentified recessive gene may influence KSHV serostatus (*15*).

The high KSHV seroprevalence (65%) among Mapuera Amerindians <10 years of age contrasts with the low (9.8%) seroprevalence among non-Amerindians of the same age group living in the same area, which suggests different transmission modes in these neighboring populations. Although we did not collect data on the age of initial sexual experience in either population, the high prevalence in childhood and inverse association with HSV-2 supports nonsexual transmission of KSHV in Amerindians. Conversely, the association of KSHV infection with HSV-2 among Mapuera non-Amerindians and blood donors supports a role for sexual transmission in these groups, although saliva transmission in younger urban inhabitants cannot be ruled out. Universal HAV infection status and low rates of HBV and HCV in all populations precluded any meaningful analysis of transmission routes associated with hepatitis viruses.

In summary, this study contributes data on the epidemiology of KSHV infection and transmission in some Brazilian Amazonian populations. Irrespective of urban or rural setting, our data are consistent with a predominant non-sexual transmission of KSHV (most likely through saliva) in Amerindian tribes compared with a probable combination of sexual and nonsexual modes of transmission among non-Amerindian populations living in the same region.
